# 

**Published:** 1889-10-12

**Authors:** 


### NOTICE TO CORRESPONDENTS.

All MS., Letters, Books for Review, and other matters intended for the Editor, should be addressed The Editor, The Lodge, Porchester Square, London, W.

### Notice to Advertisers and Subscribers.

All Advertisements, Orders for Copies of The Hospital, and Business Communications should be addressed to The Publisher, *not to the Editor*, The Hospital, 139-140, Salisbury-court, Fleet-street, E.C.

Vol. V., now ready, 4s., post free. Cases for binding, may be had of the Publisher, at 1s. 6d. each.

### To Residents, Secretaries, and Matrons.

The Editor will be glad to have the Regulations and each Report as issued by Asylums, Hospitals, Convalescent and Nursing Institutions, as well as those of other Charities, and an early copy in duplicate of all Appeals and Festival Notices, etc., addressed to *The Editor, The Lodge, Porchester-square, London, W.* Any superintendent, secretary, matron, or other chief official who regularly sends such information may be registered, on application to the Publisher, to receive free of cost a cover or binding The Hospital in half-yearly volumes.

### Scraps and Gleanings.

On November 1st a master will be elected to the Rotunda Hospital, Dublin.

A bazaar has been held at Bingley in aid of the Jubilee Cottage Hospital.

*Good Words* contains an excellent sketch of village life called "Our Old Doctor."

Messrs. Pears have taken a gold medal for their toilet soap at the Paris Exhibition.

A new Eye, Ear, and Throat Hospital has been opened at 2, North-place, Gloucester.

The report of the Royal College of Surgeons will be read at a meeting on November 7th.

Worthing Infirmary treated 24 in-patients, and 400 out-patients during September.

The death is announced of Surgeon-Major Thomas Faris, of the Army Medical Department.

A Wolverhampton butcher has been fined £25 for offering for sale meat infested with tubercles.

The Women's Medical College, of Chicago, has commenced its twentieth session with over 100 pupils.

Typhoid is decreasing in Paris, but 204 deaths occurred last week from phthisis, and 64 from cancer.

Miss Forster, of South Hetton, has given £200 towards the Hartley Wing of Sunderland Infirmary.

The Queen has given a donation of £50 to King's College Hospital, now in its fiftieth year of existence.

A meeting has been held at Bath to pass the new rules for the Eye Infirmary. Mr. H. D. Skrene presided.

Lord Rothschild has joined the Robin Society. Dinners are to be given to 5,000 children on Christmas Day.

The *Broad Arrow* of Sept. 28th contained an article on the vexatious red-tapeism which rules in military hospitals.

London for several weeks during the past few months has occupied a place among the healthiest of large British towns.

The *Nineteenth Century* contains an able article on "Mental and Physical Training of Children," by Mrs. Jessie Waller.

We see that Spratt's Patent Limited score heavily at the Paris Exhibition, receiving three awards, one being a gold medal.

The new rules at Carmarthenshire Infirmary do not please everybody, so their further consideration has been deferred till Nov. 8th.

The Hawaiian Consul is extremely indignant at the exaggerated accounts of the miseries of Molokai now going the round of the press.

The Harveian Oration will be delivered at the Royal College of Physicians on Friday, the 18th inst., at 4 p.m., by Dr. James E. Pollock.

The death is recorded of Dr. Protheroe Smith, the well-known specialist in diseases of women. He performed his first case of ovariotomy in 1843.

Mr. Jonathan Hutchinson, jun., F.R.C.S.Eng., has been elected assistant surgeon to the London Hospital, in the vacancy caused by the resignation of Mr. Couper.

Switzerland refuses to permit English doctors to practice within its boundaries in spite of overtures commenced by Lord Rosebery when he was at the Foreign Office in 1888.

Mr. Samuel Hyde, L.R.C.P., M.R.C.S.E., physician to the Peak Hydro-Thermal Establishment, Buxton, has been writing a little book on the merits of that favourite health resort.

Tunbridge Wells Hospital Saturday Fund has been divided as follows: £25 to the Eye and Ear Hospital, £25 to the Homœopathic Dispensary, and £132 10s. to the General Hospital.

Our American correspondents have been active lately. We acknowledge this week letters from Harper's Hospital, Detroit; Associated Charities, Wilmington; and the Hopital General, Ottawa.

The subject for the Fothergillian gold medal for March, 1890, is "Peripheral Neuritis," and that for March, 1891, the "Temporary and Permanent Mental Derangements which follow Surgical Procedures."

A sum of £2,000 has been presented to the University of St. Andrews for the purpose of erecting buildings and equipping a chemical laboratory in connection with the chemical chair in the United College of St. Andrews.

A public meeting was held at the Mansion House, last week, in support of Miss Sharman's Home for Orphan Girls, at West-square, Southwark, Gravesend, Tunbridge Wells, and Newton Abbot. The Lord Mayor presided.

The Lords Justices have sanctioned the expenditure of £2,000 for the acquisition of certain lands necessary for Omagh District Lunatic Asylum; also £500 for improvements at Limerick Lunatic Asylum; and a similar amount for Enniscorthy Asylum.

The Infectious Disease Notification Act (1889) comes into force throughout London on 30th of next month, and the Local Government Board has just issued a circular explaining its provisions in its application both to London and to provincial towns.

Mr. M. Colam, secretary of the Dogs' Home, issues his return for the past month, from which it appears that no fewer than 2,619 dogs were received, fed, and sheltered at the home during that month, and that nearly 400 homes were found for the more fortunate ones.

A memorandum has been sent to the divisional surgeons of the metropolitan police, by the direction of the Commissioner, requesting them in all cases of murder or manslaughter to send a report of their post-mortem examination and conclusions as to the cause of death to the Commissioner.

A magnificent orphanage has just been opened at Innsbruck. It has been built and endowed wholly at the expense of a private philanthropist, Herr von Sieberer, who gave 1,000,000 florins for the purpose. Herr von Sieberer has refused to accept any decoration or other official reward for his generosity.

